# Opicapone improves end-of-dose neuropsychiatric fluctuations in patients with Parkinson’s disease

**DOI:** 10.1016/j.prdoa.2025.100343

**Published:** 2025-05-07

**Authors:** Roberta De Fiores, Iolanda Martino, Andrea Quattrone, Basilio Vescio, Gennarina Arabia, Antonio Gambardella, Maurizio Morelli

**Affiliations:** a Institute of Neurology, Department of Medical and Surgical Sciences, Magna Graecia University, 88100 Catanzaro, Italy; b Neuroscience Research Center, Magna Graecia University, 88100 Catanzaro, Italy; c Biotecnomed S.C.aR.L., 88100 Catanzaro, Italy; d Institute of Bioimaging and Complex Biological Systems, IBSBC-CNR, Catanzaro, Italy

**Keywords:** Opicapone, Parkinson’s Disease, End-of-dose fluctuations, Neuropsychiatric fluctuations, Non-motor symptoms

## Abstract

**Introduction:**

Non-motor fluctuations (NMF) represent one of the main complications that patients with Parkinson’s disease (PD) may experience during long-term levodopa treatment. Opicapone (OPC), a COMT-inhibitor indicated for end-of-dose motor fluctuations (MF), has not been extensively investigated for the management of NMF. We evaluate the efficacy of OPC on end-of-dose neuropsychiatric fluctuations, the most frequent and severe NMF.

**Methods:**

We assessed 15 PD patients who underwent treatment with OPC (PD-OPC) compared to a control group of 15 PD patients (PD-CTRL). All patients had end-of-dose MF and NMF, confirmed by 19-item Wearing-Off Questionnaire (WOQ-19). For each, we identified the first end-of-dose deterioration period through MDS-UPDRS-III administered every 30 min over two consecutive days. On the third day, a comprehensive clinical and neuropsychological battery was administered during this designated period. Subsequently, OPC was prescribed to PD-OPC. After 6 months, patients were re-evaluated using the same baseline assessments during the same end-of-dose period.

**Results:**

At 6-month follow-up, PD-OPC showed significant improvement in the following tests: WOQ-19 (*p* < 0.001), total MDS-UPDRS and each of its four parts (*p* < 0.001), NMSS (*p* < 0.001), executive functions/attention (Weigl’s, *p* < 0.001; FAS, *p* < 0.001; FAB, *p* < 0.001; STROOP, *p* = 0.001) and mood related-symptoms (BDI-II, HAM-A; both *p* = 0.001). There was a slightly significant difference in Visual Search (*p* = 0.018), and no differences in RAVLT-I *(p* = 0.323), and RAVLT-D (*p* = 0.155) scores. At follow-up, PD-CTRL showed no significant differences in WOQ-19, motor scales, and neuropsychological tests compared to baseline.

**Conclusion:**

OPC improved end-of-dose fluctuations in anxiety/depression, and executive functions/attention, while memory and visuospatial abilities showed little or no significant changes.

## Highlights

• Non-motor fluctuations are debilitating complications in levodopa-treated PD patients.
• End-of-dose neuropsychiatric symptoms are the most common non-motor fluctuations.
• Opicapone is a COMT inhibitor indicated for end-of-dose motor fluctuations.
• Opicapone improves fluctuations in anxiety/mood and executive functions/attention.
• Opicapone is useful for the management of neuropsychiatric fluctuations in PD.

## 1 Introduction

Non-motor symptoms (NMS) comprise a variety of neuropsychiatric, sleep, autonomic, and sensory disorders that can appear during all stages of Parkinson’s disease (PD) [1]. Their prevalence remains low for the first 2 years of the disease, increasing with its progression and ultimately dominating the clinical picture of advanced PD [1,2]. Moreover, in chronic exposure to levodopa treatment, as for motor symptoms (MS), many of the NMS can fluctuate, manifesting as non-motor fluctuations (NMF) [3]. Among these, neuropsychiatric fluctuations are the most frequent and severe, having the greatest impact on caregiver burden and deterioration of quality of life [3]. Although the pathophysiology of NMF is not well known and may involve multiple neurotransmitter systems, strong evidence indicates that, similar to motor fluctuations (MF), dopaminergic mechanisms play a central role in their development [3]. Indeed, NMF are mainly associated with the off-state, occurring primarily during the end-of-dose deterioration period [3]. Therefore, continuous dopaminergic stimulation through optimizing levodopa plasma concentration represents an efficient strategy for their treatment [4]. Opicapone (OPC) is a third-generation, once-daily, peripherally selective catechol-O-methyltransferase (COMT) inhibitor that prolongs the half-life of levodopa, resulting in more stable plasma levels and, consequently, in a more continuous supply of dopamine to the brain [5]. Clinical studies [6,7] have shown that OPC enhances and extends the therapeutic effect of levodopa in PD patients with end-of-dose MF, reducing daily off-time without increasing on-time with troublesome dyskinesias. Although growing evidence suggests a potential effect of OPC on non-motor features [[8], [9], [10]], its impact on mood and cognitive fluctuations has never been investigated. Our case-control study aims to evaluate, for the first time, the efficacy of OPC on end-of-dose neuropsychiatric fluctuations in PD patients during a 6-month observation period.

## 2 Methods

We recruited two groups of PD patients with at least 3 months history of end-of-dose MF and NMF; 18 PD patients (10 male and 8 female; age at examination mean ± SD, 69.7 ± 6.6 years) underwent OPC treatment (PD-OPC group) and 17 PD patients (8 male and 9 female; age at examination, 70.6 ± 6.7 years) as controls (PD-CTLR). All PD patients referred to the Institute of Neurology, Department of Medical and Surgical Sciences, Magna Graecia University of Catanzaro, Italy. Based on the Movement Disorder Society (MDS) diagnostic criteria for PD [11], each patient had a certainty level of clinically established PD on the basis of the following features: evidence of clear and dramatic beneficial response to levodopa therapy in all patients; presence of levodopa-induced dyskinesia (LID) in 10 PD-OPC and in 11 PD-CTRL patients; rest tremor of a limb documented on clinical examination in 11 PD-OPC (including 8 patients without LID) and in 11 PD-CTRL patients (including 6 patients without LID); absence of any red flags and absolute exclusion criteria in all patients [11].

Both mean daily levodopa dose (mg/day), levodopa equivalent daily dose (LED) and mean pramipexole and ropinirole dose (mg/day) were available for each PD patient. Moreover, both PD groups showed normal scores in Mini Mental State Examination (MMSE) and Montreal Cognitive Assessment (MOCA) while involvement of smell on olfactory tests adjusted for age and sex was evident in 21 patients (10 PD-OPC and 11 PD-CTRL patients). Finally, all PD patients enrolled in the study showed abnormal striatal uptake in Dopamine Transporter Single Photon Emission Computed Tomography (DAT-SPECT) whereas decrease in myocardial uptake was observable in all 27 patients (14 PD-OPC and 13 PD-CTRL patients) who performed myocardial ^123^metaiodobenzylguanidine scintigraphy.

Exclusion criteria included treatment with other COMT inhibitors and/or neuroleptics, anxiolytics, antidepressants; thyroid disorders or other major medical illnesses, including psychiatric and/or neurological conditions that could interfere with the clinical evaluation; unpredictable off-state periods; clinical and/or radiological features suggestive of atypical parkinsonism; a clinical diagnosis of dementia; evidence of magnetic resonance imaging abnormalities such as lacunar infarctions in the basal ganglia and/or subcortical with diffuse periventricular signal alterations; normal striatal uptake in DAT-SPECT. All study procedures and ethical aspects were approved by the institutional review board of Magna Graecia University of Catanzaro, Italy, and the study was conducted in accordance with the Code of Ethics of the World Medical Association (Declaration of Helsinki) for experiments involving humans. Written informed consent was obtained from all patients at the time of study recruitment. After the enrolment, both group of PD patients underwent a baseline and 6-month follow-up clinical evaluation. A week before the baseline clinical assessment, the diagnosis of end-of-dose MF and NMF was confirmed using the validated Italian version of the 19-item Wearing-Off Questionnaire (WOQ-19) [12,13] in which each patient self-reported the presence of any of the indicated 9 MS and/or 10 NMS experienced during the day, and whether these symptoms improved after the next scheduled dose of levodopa [12,13]. A cut-off of at least two symptoms fluctuating with medication intake was applied [13], with at least one fluctuating symptom in the motor domain and one in the non-motor domain. Therefore, the baseline clinical evaluation was performed over three consecutive days. On the first two days, a movement disorders specialist assessed each patient using the validated Italian version of the MDS-Unified Parkinson’s Disease Rating Scale part III (MDS-UPDRS-III) [14,15] every 30 min, starting from the first-morning dose of levodopa. The end-of-dose deterioration period was identified as the time when the MDS-UPDRS-III score dropped by at least 30 % compared to the ON state, with subsequent improvement following the intake of antiparkinsonian medication [16]. On the third day, the same neurologist re-evaluated only those patients who had shown end-of-dose deterioration periods at the same time during the previous two days. The assessment was performed at the first end-of-dose deterioration time of the day, using the following clinical scales: the validated Italian version of MDS-UPDRS for disease extent and burden [14,15], the Hoehn and Yahr (H-Y) rating scale for disease severity [17], and the Non-Motor Symptoms Scale (NMSS) for the assessment of NMS [18]. Immediately after the neurological evaluation, a trained neuropsychologist evaluated each patient using the following tests: Weigl’s test [19], FAS test [20], Frontal Assessment Battery (FAB) [21], Stroop test [22], Visual Search [23], Rey Auditory Verbal Learning Test – Immediate Recall (RAVLT-I) [24], RAVLT − Delayed Recall (RAVLT-D) [24] Beck's depression inventory-II (BDI-II) [25], and Hamilton Anxiety Rating Scale (HAM-A) [26]. After completing all clinical and neuropsychological evaluations, patients received their second daily dose of antiparkinsonian medication, followed by an MDS-UPDRS-III assessment [14,15] to confirm post-dose improvement. At the end of enrolment, each patient in PD-OPC group was prescribed oral OPC (50 mg) to be taken once daily at bedtime, at least 1 h after the last daily dose of levodopa, and away from meals [5]. No changes to patients’ antiparkinsonian therapy were allowed during the follow-up period in both groups; if therapeutic adjustments were made, the patient was excluded from the study. A week before the 6-month follow-up assessment, all patients completed the WOQ-19 again [12,13]. At the 6-month follow-up evaluation, each patient was re-assessed at the same time of day when end-of-dose phenomena had been observed at baseline. The same neurologist and neuropsychologist conducted the re-evaluation using the same clinical and neuropsychological scales.

## 3 Statistical analyses

Sex differences between PD-OPC and PD-CTRL groups were evaluated by means of Fisher’s exact test. The Shapiro-Wilk test was used to check the distribution of variables at baseline evaluation, in order to choose the most appropriate test for comparison: age at baseline evaluation, age at disease onset, mean daily levodopa dose, LED and MOCA were compared using Student’s *t*-test, while differences in disease duration, pramipexole dose, ropinirole dose and MMSE were assessed using Wilcoxon’s rank sum test. As two groups of PD patients were observed in two different time points, differences between PD-OPC and PD-CTRL groups at baseline and follow-up evaluations and differences within groups, between baseline and follow-up evaluations, were assessed for motor and non-motor scales and for scores from neuropsychological tests by means of mixed model ANOVA tests, with p values corrected according to Tukey method. All statistical analyses were performed using the R statistical software, version 4.2.2. (2022, The R foundation for Statistical Computing).

## 4 Results

Of the initial PD patients enrolled, 15 PD-OPC and 15 PD-CTRL patients completed the 6-month observation period. Five patients were excluded: three dropped out, and two did not exhibit end-of-dose deterioration periods simultaneously on two consecutive days during the baseline evaluation. No patient required any changes to their therapy throughout the entire follow-up period.

At baseline evaluation, no significant differences (Supplementary Table 1) were evident between PD-OPC and PD-CTRL groups in sex (*p* = 1); age at examination (*p* = 0.739); age at disease onset (*p* = 0.811), disease duration (*p* = 0.393), mean daily levodopa dose (*p* = 0.754); LED (*p* = 0.852); mean daily pramipexole (*p* = 0.614) and ropinirole dose (p = 0.905), MMSE (*p* = 0.224) and MOCA (p = 0.799).

At the 6-month follow-up evaluation, the mean number of symptoms reported on the WOQ-19 was significantly reduced (p < 0.001) in PD-OPC group (Table 1). The decrease was also maintained when considering MS and NMS (both p < 0.001) separately. A similar trend was observed for symptoms that improved with medication intake. At baseline, using the WOQ-19, each patient self-reported at least two fluctuating symptoms (mean ± SD, 8.6 ± 1.7) (Table 1). After six months of OPC therapy, the mean number of these symptoms showed a significant decrease (p < 0.001), both overall (p < 0.001) and for the two components of MS and NMS (both p < 0.001). Instead, PD-CTRL showed no significant differences at follow-up both in experienced symptoms (total score, *p* = 0.128; MS, *p* = 0.383 and NMS, *p* = 0.346) and in symptoms that improve after the next dose (total score, *p* = 0.518; MS, *p* = 0.449 and NMS, *p* = 0.405) on the WOQ-19 (Table 1).

**Table 1:** Changes in WOQ-19 scores at baseline and after 6 months in patients with Parkinson disease in treatment with opicapone (PD-OPC) and in patients with Parkinson disease without treatment with opicapone (PD-CTRL).

WOQ-19^a^		**Experience symptoms**			**Symptoms that improve** **after the next dose**		
		Total score	Motor symptoms score	Non-motor symptoms score	Total score	Motor symptoms score	Non-motor symptoms score
**PD-OPC**	Baseline	11.5 ± 1.9	5.3 ± 1.1	6.2 ± 1.1	8.6 ± 1.7	4.1 ± 0.9	4.5 ± 1.0

Follow-up	9.0 ± 1.7	4.1 ± 1.0	4.9 ± 0.9	5.9 ± 1.6	2.7 ± 0.8	3.2 ± 1.0
**PD-CTRL**	Baseline	11.0 ± 2.1	5.2 ± 0.9	6.0 ± 1.0	8.7 ± 1.6	4.3 ± 0.9	4.7 ± 0.8
	Follow-up	11.9 ± 1.9	5.5 ± 1.2	6.3 ± 1.0	9.1 ± 1.4	4.7 ± 0.6	5.0 ± 0.8
***P* value^b^**	***Intragroup comparison (baseline vs. follow-up)***						
	PD-OPC *vs.* PD-OPC	<0.001	<0.001	<0.001	<0.001	<0.001	<0.001
	PD-Control *vs.* PD-Control	0.128	0.383	0.346	0.518	0.449	0.405
	***Intergroup comparison (PD-OPC vs. PD-Control)***						
	Baseline *vs.* baseline	0.908	0.998	0.947	0.996	0.826	0.979
	Follow-up *vs.* follow-up	0.001	0.003	0.003	<0.001	<0.001	<0.001

a Values are expressed as mean ± Standard Deviation. ^b^Mixed model ANOVA, followed by Tukey HSD test. Abbreviations: WOQ-19, validated Italian version 19-item Wearing-Off Questionnaire; PD-OPC, patients with Parkinson disease in treatment with opicapone; PD-CTRL, patients with Parkinson disease without treatment with opicapone.

Furthermore, after 6 months of OPC treatment, there was a statistically significant reduction in the total MDS-UPDRS (*p <* 0.001) and in each of its four parts: part I – non-motor aspect of experiences of daily living, *p* < 0.001; part II – motor aspect of daily living, *p* < 0.001; part III – motor examination, *p* < 0.001 and part IV – motor complications, *p* < 0.001 (Table 2). As regards the two sections of part IV, section A (dyskinesias) showed a slight and non-significant score increase (*p* = 0.695), while section B (motor fluctuations) demonstrated a significant score reduction (*p* < 0.001) (Table 2). The H-Y rating scale values remained stable throughout the six-month observation period (*p* = 0.947) (Table 2). Moreover, there was a significant reduction in NMSS total score at follow-up evaluation (*p* < 0.001) with significant improvements in the two non-motor domains of NMSS: Domain 3 − Mood/Cognition (*p* < 0.001) and Domain 5 − Attention/Memory (*p* < 0.001) (Table 2).

**Table 2:** Changes in MDS-UPDRS, H-Y rating scale, and NMSS scores at baseline and at 6-month follow-up in patients with Parkinson disease in treatment with opicapone (PD-OPC) and in patients with Parkinson disease without treatment with opicapone (PD-CTRL).

Test*		**MDS-UPDRS score**							**H-Y scale score**	**NMSS score**		
		**Total**	**Part I** **(nM-EDL)**	**Part II** **(M−EDL)**	**Part III (ME)**	**Part IV (MC)**	**Part IV-A (DYS)**	**Part IV-B (MF)**		**Total**	**Domain 3 Mood/Cognition**	**Domain 5 Attention/memory**
**PD-OPC**	**Baseline**	79.3 ± 8.5	15.8 ± 2.3	19.1 ± 2.9	36.1 ± 6.2	8.2 ± 1.7	1.0 ± 0.9	7.2 ± 1.1	2.2 ± 0.5	76.1 ± 26.1	26.5 ± 8.8	8.4 ± 2.9

**Follow-up**	59.8 ± 10.0	11.8 ± 2.3	14.8 ± 2.8	27.2 ± 6.6	6.1 ± 1.6	1.2 ± 1.0	4.8 ± 1.2	2.2 ± 0.3	58.1 ± 22.4	19.6 ± 6.6	6.5 ± 1.9
**PD-CTRL**	**Baseline**	80.1 ± 9.6	15.9 ± 1.4	18.9 ± 1.8	37.5 ± 4.3	8.3 ± 1.6	1.1 ± 0.8	7.5 ± 0.7	2.5 ± 0.5	79.7 ± 20.3	23.4 ± 7.4	9.0 ± 1.6
	**Follow-up**	83.0 ± 7.4	16.3 ± 1.5	19.7 ± 1.4	38.7 ± 4.1	8.6 ± 1.1	1.5 ± 0.5	7.9 ± 1.1	2.8 ± 0.7	84.8 ± 17.3	23.7 ± 6.7	9.3 ± 1.3
*P* value**	***Intragroup comparison****(baseline vs. follow-up)*											
	PD-OPC *vs.* PD-OPC	<0.001	<0.001	< 0.001	<0.001	<0.001	0.695	<0.001	0.947	<0.001	<0.001	<0.001
	PD-CTRL *vs.* PD-CTRL	0.108	0.569	0.171	0.245	0.332	0.151	0.410	0.050	0.141	0.951	0.861
	***Intergroup comparison****(PD-OPC vs. PD-CTRL)*											
	Baseline *vs.* baseline	0.996	1.000	0.995	0.907	1.000	0.972	0.952	0.726	0.970	0.671	0.852
	Follow-up *vs.* follow-up	<0.001	<0.001	<0.001	<0.001	<0.001	0.702	<0.001	0.016	0.011	0.433	0.004

* Data are expressed as mean ± Standard Deviation; **Mixed model ANOVA, followed by Tukey HSD test. Abbreviations: MDS-UPDRS, Italian version of Movement Disorder Society-Unified Parkinson’s Disease Rating Scale; H-Y, Hoehn-Yahr; NMSS, Non-Motor Symptoms Scale; nM-EDL, Non-Motor Aspects of Experiences of Daily Living; M−EDL, Motor Aspects of Experiences of Daily Living; ME, Motor Examination; MC, Motor Complications; DYS, Dyskinesias; MF, Motor Fluctuations; PD-OPC, patients with Parkinson disease in treatment with opicapone; PD-CTRL, patients with Parkinson disease without treatment with opicapone.

By contrast, total MDS-UPDRS, each of the four parts of MDS-UPDRS, H-Y rating scale and NMSS (total score, domain 3 and domain 5) showed no significant differences in PD-CTRL between baseline and 6-month follow-up evaluation (Table 2).

**Fig. 1:**
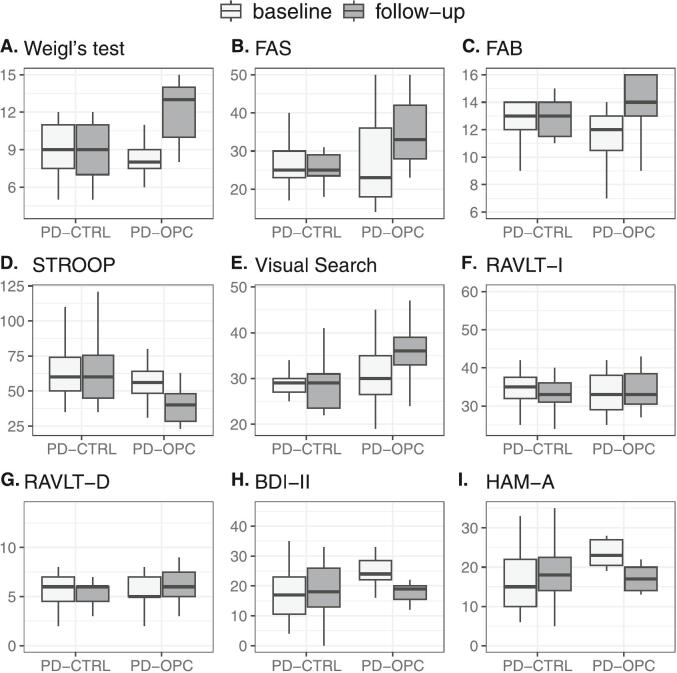
Boxplots of neuropsychological tests at baseline and follow-up in patients with Parkinson disease in treatment with opicapone (PD-OPC) and in patients with Parkinson disease without treatment with Opicapone (PD-CTRL). A. Weigl’s test; B. FAS Fluency test; C. Frontal Assessment Battery (FAB); D. STROOP test; E. Visual Search test; F. Rey Auditory Verbal Learning Test (RAVLT)-Immediate recall; G. RAVLT-Delayed recall; H. Beck’s Depression Inventory; I. Hamilton Anxiety Rating Scale. Each box's lower and upper lines describe the 25th and 75th percentile, respectively; the bold line within the box represents the median (50th percentile), while the lower and upper hinge of whiskers represent the minimum and maximum value, respectively; outliers are depicted as dots.

The neuropsychological scores at baseline and after 6 months of OPC treatment are reported in Table 3 and Fig. 1. At the 6-month follow-up, we observed a significant increase in the scores of Weigl’s test (*p* < 0.001), FAS Fluency test (*p* < 0.001) and FAB (*p* < 0.001), along with a significant reduction in the scores of STROOP test (*p* = 0.001), BDI-II (*p* = 0.001), and HAM-A (*p* = 0.001) (Table 3, Fig. 1) compared to baseline. In contrast, there was a slightly significant difference between baseline and follow-up in the scores of Visual Search (*p* = 0.018), and no significant differences in RAVLT-I *(p* = 0.323), and RAVLT-D (*p* = 0.155) (Table 3, Fig. 1).

**Table 3:** Changes in neuropsychological tests at baseline and after 6 months in patients with Parkinson disease in treatment with opicapone (PD-OPC) and in patients with Parkinson disease without treatment with opicapone (PD-CTRL).

**Test**^a^		**Weigl’s test**	**FAS Fluency test**	**FAB**	**STROOP test**	**Visual search**	**RAVLT-I**	**RAVLT-D**	**BDI-II**	**HAM-A**
**PD-OPC**	**Baseline**	8.2 ± 2.0	26.1 ± 11.0	11.7 ± 2.2	56.1 ± 12.3	31.4 ± 7.7	33.8 ± 5.4	5.5 ± 1.6	25.1 ± 4.9	23.3 ± 3.4

**Follow-up**	12.1 ± 2.4	35.1 ± 8.9	13.7 ± 2.7	41.7 ± 17.3	37.1 ± 6.7	35.6 ± 8.2	6.3 ± 2.9	17.8 ± 3.1	17.2 ± 3.3
**PD-CTRL**	**Baseline**	8.8 ± 2.3	26.7 ± 6.4	12.5 ± 2.0	61.7 ± 19.7	28.7 ± 4.1	34.6 ± 4.4	5.7 ± 1.8	18.6 ± 10.9	17.1 ± 8.2
	**Follow-up**	8.9 ± 2.3	26.7 ± 6.2	11.9 ± 2.9	63.5 ± 24.0	28.7 ± 5.7	33.0 ± 4.1	4.9 ± 1.8	19.4 ± 12.3	17.8 ± 9.3
***P* value^b^**	***Intragroup comparison (baseline vs. follow-up)***									
	PD-OPC *vs.* PD-OPC	<0.001	<0.001	< 0.001	0.001	0.018	0.323	0.155	0.001	0.001
	PD-CTRL *vs.* PD-CTRL	0.978	1.000	0.433	0.959	1.000	0.425	0.276	0.962	0.956
	***Intergroup comparison (PD-OPC vs. PD-CTRL)***									
	Baseline *vs.* baseline	0.887	0.998	0.772	0.847	0.643	0.981	0.993	0.195	0.067
	Follow-up *vs.* follow-up	0.003	0.047	0.209	0.016	0.003	0.609	0.265	0.958	0.995

a Values are expressed as mean ± Standard Deviation. ^b^Mixed model ANOVA, followed by Tukey HSD test. Abbreviations: FAB, Frontal Assessment Battery; RAVLT-I, Rey Auditory Verbal Learning Test − Immediate Recall; RAVLT-D, Rey Auditory Verbal Learning Test − Delayed Recall; BDI-II, Beck Depression Inventory-II; HAM-A, Hamilton Anxiety Rating Scale. WOQ-19, validated Italian version 19-item Wearing-Off Questionnaire; PD-OPC, patients with Parkinson disease in treatment with opicapone; PD-CTRL, patients with Parkinson disease without treatment with opicapone.

Instead, no significant change in the scores of any neuropsychological tests in PD-CTRL group was evident between baseline and 6-month follow-up evaluation (Table 3; Fig. 1).

## 5 Discussion

In this 6-month follow-up study, we investigated, for the first time, the effect of OPC treatment on end-of-dose neuropsychiatric fluctuations in PD patients. At the end of the observational period, the addition of OPC to levodopa therapy led to significant improvements in mood and cognitive fluctuations, besides confirming its positive effect on end-of-dose MF.

MF and NMF represent one of the main complications that patients undergoing long-term levodopa treatment may experience as PD advances [3,27]. The development of MF is associated with non-physiological, pulsatile stimulation of dopamine receptors in the striatum [3]. Indeed, as dopaminergic neurons in the substantia nigra degenerate, their buffering capacity is progressively lost, leading nigrostriatal dopamine levels to become increasingly dependent on the peripheral availability of levodopa [3,4,28]. In particular, end-of-dose deterioration, also known as wearing-off, is linked to the progressive reduction in the effect of oral levodopa, which already has a short half-life [4,28]. By inhibiting the peripheral COMT enzyme, OPC prolongs levodopa’s half-life and enhances its bioavailability, thereby providing less pulsatile and more continuous dopaminergic stimulation in the central nervous system [5]. While the enhanced bioavailability of levodopa improves MF, it may conversely induce dyskinesias, which are the most common adverse event related to OPC [5]. The pivotal phase 3 BIPARK 16 and BIPARK 27 trials, along with their open-label extensions, demonstrated the lasting effectiveness of OPC in reducing end-of-dose MF without increasing on time with troublesome dyskinesias. Our findings are consistent with this evidence, in fact, after 6 months of OPC treatment, we observed a significant reduction in the mean number of MS fluctuating with medication intake, as evidenced by the WOQ-19. Likewise, the scores for part II of MDS-UPDRS, which assesses motor experiences of daily living, and part III, which concerns the motor examination, both evaluated during the end-of-dose deterioration time, showed a statistically significant reduction. Finally, our data showed a slight and not statistically significant increase in the score of MDS-UPDRS part IV, section B (dyskinesias).

Alongside the well-established data confirming the OPC effect on end-of-dose MF, less robust evidence regarding its impact on non-motor features, especially NMF, is available [[8], [9], [10]]. In the present study, after 6 months of OPC treatment, we observed a significant reduction in the mean number of NMS that improved after the next dose of levodopa, as self-reported by patients through WOQ-19. The effectiveness in controlling NMF was further confirmed by the significant reduction in scores on the following non-motor scales, both evaluated during the end-of-dose deterioration period: part I of the MDS-UPDRS, which assessed non-motor aspect of experiences of daily living, and NMSS, which reflects the global NMS burden. Furthermore, when evaluating the single domains of the NMSS at the end-of-dose deterioration time, for those related to neuropsychiatric symptoms, the decrease was sustained. Specifically, a significant reduction was observed in domains 3 and 5 of NMSS, which assess mood/cognition and attention/memory, respectively. The same applied in the neuropsychological assessments, where significant improvements were noted in the scores of executive functions and attention tests, including Weigl’s test, FAS fluency test, FAB, and STROOP test. Scores on tests evaluating visuospatial abilities, such as the Visual Search test, and verbal memory (both immediate and delayed), such as RAVLT-I and RAVLT-D, showed little or no statistically significant changes. Finally, a significant improvement was also clearly evident in the scores for anxiety and depression, as measured by the HAM-A and BDI-II, respectively. These results, demonstrating an improvement in fluctuating cognitive and mood symptoms following OPC treatment, are particularly significant since neuropsychiatric fluctuations are the most common and severe NMF, responsible for considerable disability and deterioration of quality of life for both PD patient and their caregiver [3]. Despite the considerable impact on disease burden, their prevalence is underestimated, and many aspects remain poorly understood, including their pathophysiology and management [3]. Evidence suggests that cognitive and mood fluctuations in PD patients involve multiple neurotransmitters, including dopamine, acetylcholine, norepinephrine, and serotonin [3,27,29]. However, degeneration of the dopaminergic system remains the primary driver, with the cell death of dopaminergic mesocortical and mesolimbic neurons appearing to be the main substrate [3,27,29]. Actually, the pathophysiological mechanism is significantly more complex and involves the prefrontal cortex (PFC), caudate nucleus, nucleus accumbens, and many other structures, often modulated by dopamine [30]. More specifically, impairments in executive functions and their fluctuations in PD patients appear to rely on abnormal dopaminergic modulation within the frontostriatal circuit linking dorsolateral PFC to the caudate nucleus, the substantia nigra and the globus pallidus, and the thalamus [31]. The similarity between the working memory deficits caused by dopamine depletion from the PFC and those resulting from complete ablation of the PFC confirms the key role of dopamine in the above-mentioned circuits [32]. Studies demonstrating the beneficial effects of levodopa on working memory, planning, and certain types of learning further reinforced these findings [29]. Likewise, dopamine's involvement in the cortical-subcortical circuits that regulate emotions, motivation, and reward is crucial for the development of anxiety and mood fluctuations observed in PD patients [32]. This is supported by studies showing that when dopamine levels in the mesolimbic system are at their lowest, PD patients experience a state of neuropsychiatric hypoactivity, with depression, anxiety, and lack of motivation [3]. Complementarily, additional evidence is provided by the significant reduction in depression levels following treatment with dopaminergic drugs that have a high affinity for D2/D3 receptors, those with the highest expression levels in the limbic system [3,33].

To summarize, the available laboratory and clinical evidence indicate that dopamine plays a key role in the fluctuations of anxiety, depression, and cognitive functions in PD patients [3].

This could explain why OPC effectively alleviates fluctuations in anxiety, depression, executive functions, and attention in PD patients by increasing the bioavailability of levodopa and reducing daily off-time.

There are some limitations to this study. First, patients in our study had a relatively short disease duration, and none of them presented cognitive decline or hallucinations. Given that previous evidence [[5], [6], [7]] suggests that OPC may slightly increase the risk of hallucinations, we cannot exclude that prolonged treatment with OPC in PD patients with longer disease duration and/or cognitive decline might lead to the emergence of such treatment-related adverse effects. Second, the small sample size makes the study susceptible to Type II errors, due to low statistical power. Third, the study's follow-up period is limited to 6 months, leaving it unclear whether the observed effects are sustained over time. Thus, larger studies with longer follow-up times are needed. Finally, we cannot exclude a potential influence on cognition and mood by other antiparkinsonian drugs. However, the mean dosages of antiparkinsonian drugs were not significantly different between the two PD groups at baseline and none of the PD patients changed any of their therapy during the follow-up.

In our study, we demonstrated the efficacy of a 6-month treatment with OPC as an adjunct to levodopa therapy in improving end-of-dose neuropsychiatric fluctuations in PD patients. This suggests that OPC provides benefits not only for patients with MF but also in managing a disabling and common complication such as neuropsychiatric fluctuations, especially concerning anxiety, depression, and executive functions/attention.

## Author contributions

Roberta De Fiores: conception and design of the study, acquisition of data, analysis and interpretation of data, drafting the article, critical revision of the article for important intellectual content, final approval of the version to be submitted.

Iolanda Martino: acquisition of data, critical revision of the article for important intellectual content, final approval of the version to be submitted.

Andrea Quattrone: acquisition of data, critical revision of the article for important intellectual content, final approval of the version to be submitted.

Basilio Vescio: analysis and interpretation of data, critical revision of the article for important intellectual content, final approval of the version to be submitted.

Gennarina Arabia: acquisition of data, critical revision of the article for important intellectual content, final approval of the version to be submitted.

Antonio Gambardella: acquisition of data, critical revision of the article for important intellectual content, final approval of the version to be submitted.

Maurizio Morelli: conception and design of the study, acquisition of data, analysis and interpretation of data, critical revision of the article for important intellectual content, final approval of the version to be submitted.

## 7 Funding Sources

This research did not receive any specific grant from funding agencies in the public, commercial, or not-for-profit sectors.

All authors declare that this paper has not been published previously and that it is not under consideration for publication elsewhere.

All authors declare that this publication is approved by all authors and tacitly or explicitly by the responsible authorities where the work was carried out, and that, if accepted, it will not be published elsewhere in the same form, in English or any other language, including electronically without the written consent of the copyright-holder.

All authors declare that all co-authors have read and approved the submitted manuscript.

## CRediT authorship contribution statement

**Roberta De Fiores:** Writing – review & editing, Writing – original draft, Supervision, Methodology, Investigation, Formal analysis, Data curation, Conceptualization. **Iolanda Martino:** Writing – review & editing, Visualization, Data curation. **Andrea Quattrone:** Writing – review & editing, Visualization, Data curation. **Basilio Vescio:** Writing – review & editing, Visualization, Methodology, Formal analysis, Data curation. **Gennarina Arabia:** Writing – review & editing, Visualization, Data curation. **Antonio Gambardella:** Writing – review & editing, Visualization, Data curation. **Maurizio Morelli:** Writing – review & editing, Supervision, Methodology, Formal analysis, Data curation, Conceptualization.

## Declaration of competing interest

The authors declare that they have no known competing financial interests or personal relationships that could have appeared to influence the work reported in this paper.
