# Supplementary material for: Opicapone improves end-of-dose neuropsychiatric fluctuations in patients with Parkinson’s disease
Source: Clin Park Relat Disord. 2025 May 7;12:100343. doi: 10.1016/j.prdoa.2025.100343 (PMC12141878; doi:10.1016/j.prdoa.2025.100343)
Supplement: Supplementary Data 1 [file mmc1.docx]

**Supplementary table 1.** Demographic and clinical features of PD patients who will take opicapone (PD-OPC) and PD patients who will not take opicapone (PD-CTRL) at baseline evaluation.

|  | **PD-OPC**  **(*n* = 15)** | **PD-CTRL**  ***(n* = 15)** | ***P* value** |  |
| --- | --- | --- | --- | --- |
| Sex (male/female) | | 8/7 | 7/8 | 1^a^ |
| Age at examination, years^b^ | | 69.5 ± 7.1 | 70.3 ± 7.1 | 0.739^c^ |
| Age at disease onset, years^b^ | | 62.3 ± 6.8 | 62.9 ± 6.8 | 0.811^c^ |
| Disease duration, years^b^ | | 7.2 ± 1.7 | 7.5 ± 1.2 | 0.393^d^ |
| Daily levodopa dose (mg/d)^b^ | | 613.3 ± 222.4 | 640.0 ± 238.4 | 0.754^c^ |
| LED^b^ | | 809.8 ± 248.7 | 825.8 ± 216.4 | 0.852^c^ |
| Daily pramipexole dose (mg/d) | | 1.0 ± 0.6 | 1.2 ± 0.7 | 0.614^d^ |
| Daily ropinirole dose (mg/d) | | 2.8 ± 1.1 | 3.6 ± 2.6 | 0.905^d^ |
| MMSE^b^ | | 27.1 ± 1.3 | 26.5 ± 1.2 | 0.224^d^ |
| MoCA^b^ | | 25.0 ± 2.3 | 25.2 ± 1.9 | 0.799^c^ |

^a^ Fisher’s exact test; ^b^ Values are expressed as mean ± Standard Deviation; ^c^ Student’s t-test; ^d^ Wilcoxon rank sum test.

Abbreviations: PD, Parkinson’s Disease; OPC, Opicapone; CTRL, Controls; LED, Levodopa Equivalent Dose; MMSE, Mini Mental State Examination; MoCA, Montreal Cognitive Assessment.
